# Interaction Dynamics of Plant-Specific Insert Domains from *Cynara cardunculus*: A Study of Homo- and Heterodimer Formation

**DOI:** 10.3390/molecules29215139

**Published:** 2024-10-30

**Authors:** Miguel Sampaio, Sofia Santos, Ana Marta Jesus, José Pissarra, Gian Pietro Di Sansebastiano, Jonas Alvim, Cláudia Pereira

**Affiliations:** 1 GreenUPorto—Sustainable Agrifood Production Research Centre, INOV4AGRO—Institute for Innovation, Training and Sustainability of Agrifood Production & Department of Biology, Faculty of Sciences, University of Porto, Rua do Campo Alegre s/n, 4169-007 Porto, Portugal; up201503801@edu.fc.up.pt (M.S.); soliveirasofia13@gmail.com (S.S.); up201905096@edu.fc.up.pt (A.M.J.); jpissarr@fc.up.pt (J.P.); 2 DiSTeBA (Department of Biological and Environmental Sciences and Technologies), University of Salento, Via Lecce-Monteroni, Campus ECOTEKNE, 73100 Lecce, Italy; gp.disansebastiano@unisalento.it; 3 Laboratory of Plant Physiology and Biophysics, Bower Building, University of Glasgow, Glasgow G12 8SU, UK; jonas.alvim@glasgow.ac.uk

**Keywords:** plant-specific inserts (PSIs), dimerization dynamics, membrane-related processes, FRET-FLIM assays, *Cynara cardunculus*

## Abstract

Plant aspartic proteinases (APs) from *Cynara cardunculus* feature unique plant-specific insert (PSI) domains, which serve as essential vacuolar sorting determinants, mediating the transport of proteins to the vacuole. Although their role in vacuolar trafficking is well established, the exact molecular mechanisms that regulate PSI interactions and functions remain largely unknown. This study explores the ability of PSI A and PSI B to form homo- and heterodimers using a combination of pull-down assays, the mating-based split-ubiquitin system (mbSUS), and FRET-FLIM analyses. Pull-down assays provided preliminary evidence of potential PSI homo- and heterodimer formation. This was conclusively validated by the more robust in vivo mbSUS and FRET-FLIM assays, which clearly demonstrated the formation of both homo- and heterodimers between PSI A and PSI B within cellular environments. These findings suggest that PSI dimerization is related to their broader functional role, particularly in protein trafficking. Results open new avenues for future research to explore the full extent of PSI dimerization and its implications in plant cellular processes.

## 1. Introduction

Some plant aspartic proteinases (APs), unlike their animal counterparts, contain a unique domain of approximately 100 amino acids in their C-terminal region, known as the plant-specific insert (PSI). This domain has garnered attention because it is exclusive to a select group of plant APs [1] and shares structural similarities with saposin-like proteins (SAPLIPs) [2,3], suggesting key roles in membrane interactions and protein trafficking. PSIs have been associated with various in vitro functions, including interactions with lipid membranes [4], antimicrobial activity [5,6], and membrane permeabilization [7,8,9]. However, the in vivo significance of these activities, particularly their relationship to protein transport and cellular localization, remains unclear. One of the most critical roles proposed for PSIs is their function as vacuolar sorting signals, targeting APs to the vacuole in tissues with high metabolic activity, such as flowers and seeds [10,11,12,13,14]. Structurally, PSIs consist of helical domains with a loop that projects outwards [15,16], and many PSIs contain N-glycosylation sites, accessible during translocation, which help stabilizing the proenzyme and protect it from premature degradation during transport [14].

Several studies have focused on the pH-dependent membrane interaction abilities of PSIs and there is growing evidence that dimerization—both homo- and heterodimer formation—may be a critical aspect of their function. In some cases, the dimerization of PSIs has been shown to be influenced by pH, with reports of PSI domains transitioning from monomers to dimers under specific pH conditions [7,15]. For instance, the PSI from Solanum tuberosum (potato) exhibits pH-dependent activities, including dimerization, which suggests that environmental factors such as pH may regulate its interactions [17]. However, while these studies have contributed to our understanding of PSIs, the exact relationship between dimerization and functional outcomes such as their role in protein trafficking remains underexplored.

In *Cynara cardunculus*, the two best-studied APs—cardosin A and cardosin B—show intriguing differences in subcellular localization, with cardosin A being stored in the vacuole and cardosin B localizing in the extracellular matrix [18,19,20]. Further investigation has revealed that PSI domains from these APs (PSI A from cardosin A and PSI B from cardosin B) are responsible for determining the different vacuolar transport routes of these proteins: PSI A follows an unconventional Golgi-bypassing pathway, while PSI B traffics through the Golgi apparatus [10]. Moreover, an N-glycosylation site present in PSI B but absent in PSI A may be one of the factors influencing these distinct routes [14].

Given these differential trafficking routes and the structural roles of PSI domains, we hypothesize that dimerization of PSI A and PSI B could be critical for their function in vacuolar targeting and trafficking. In this study, we aim to explore the ability of PSI A and PSI B to form homo- and heterodimers and assess how these interactions contribute to their roles in vacuolar transport. While previous in vitro studies suggested pH-dependent dimerization, our focus shifts to confirming these interactions in vivo using robust techniques such as the mating-based split-ubiquitin system (mbSUS) and FRET-FLIM assays. Ultimately, this study seeks to provide new insights into the dimerization dynamics of PSI A and PSI B and to prove that dimerization processes of thistle PSIs may be of high relevance to trafficking mechanisms.

## 2. Results

### 2.1. Exploratory In Vitro Analysis of PSI Homo- and Heterodimerization Under Different pH Conditions

**Figure 1:**
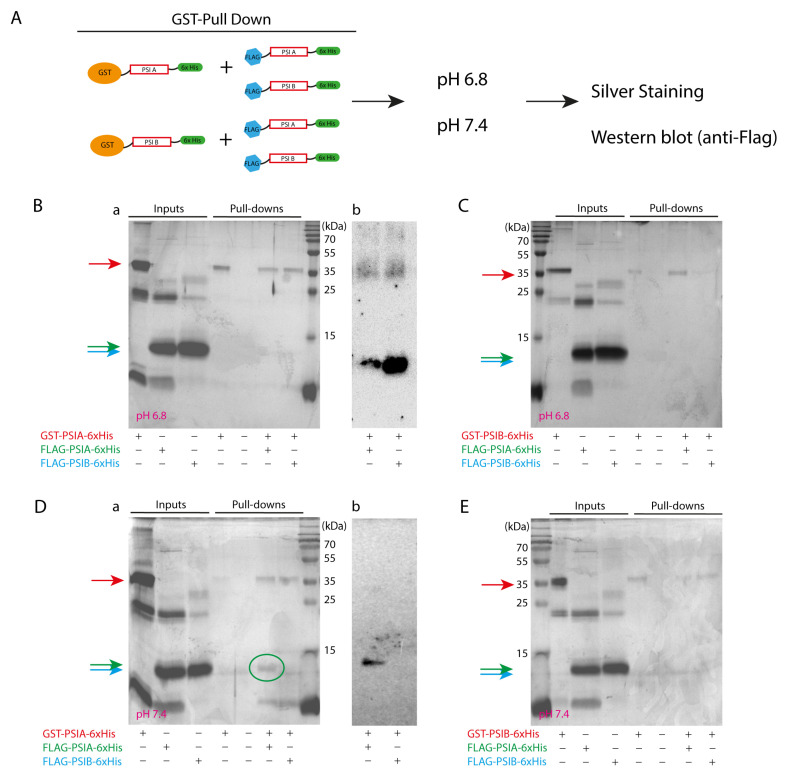
(**A**) Schematic representation of PSI-tagged versions used in pull-down assays. SDS-PAGE analysis of PSI–PSI interactions by pull-down assay at pH 6.8 (**B**,**C**) and 7.4 (**D**,**E**). (**B**(**a**),**C,D**(**a**) and **E**) Inputs and pull-down reactions were analyzed on a silver-stained gel. Each pull-down lane was loaded with 15 μL of pull-down reaction and input lanes were loaded with 1/3 of the amount of purified protein added to each reaction. (**B**)(**b**)–(**D**)(**b**) Western blot analysis to evaluate the presence of FLAG-tagged PSIs in the pull-down reactions. MW: Molecular Weight (PageRuler^TM^ Prestained Protein Ladder, Thermo Scientific, Waltham, MA, USA). Red, green, and blue arrows represent GST-PSIA/PSIB-6xHis, FLAG-PSI A-6xHis, and FLAG-PSIB-6xhis, respectively.

To initially explore the potential interactions between PSI A and PSI B, a pull-down assay was conducted under two pH conditions: 6.8 and 7.4. The purified PSIs—GST-PSI (A/B)-6xHis and FLAG-PSI (A/B)-6×His—were obtained by batch purification from bacterial lysates using HisPur Ni-NTA Superflow Agarose (Supplementary Figure S1). The presence of the PSIs in various fractions was evaluated by Coomassie blue staining, and the expected band sizes of ~35 kDa for GST-tagged PSIs and ~10–15 kDa for FLAG-tagged PSIs were detected in all relevant fractions. These pH values (6.8 and 7.4) were selected to ensure efficient binding of GST-tagged proteins, which typically exhibit suboptimal binding outside the pH range of 6.5–8. In this exploratory setup, PSIs fused to GST tags served as bait to capture possible interactors (Figure 1A). At pH 6.8, input lanes showed bands of the correct size for both GST-PSI (A/B) and FLAG-PSI (A/B) (Figure 1B(a),C). In the pull-down reactions, GST-tagged PSI proteins bound successfully to the glutathione–agarose beads, with no detectable nonspecific binding of FLAG-tagged proteins. However, only GST-fused PSIs were observed in the pull-down lanes, with no clear evidence of FLAG-PSI A or B interactions at this pH, suggesting limited or no interaction under these conditions. To clarify, a Western blot using an anti-FLAG antibody was used in these samples, and indeed, at pH 6.8, FLAG-PSI A and FLAG-PSI B were detected, indicating interactions between PSI A and PSI B (Figure 1B(b)). At pH 7.4, input lanes again confirmed the correct presence of GST-PSI A and B and FLAG-PSI A and B (Figure 1D(a),E). While both GST-fused PSIs were observed across all pull-down lanes, a distinct interaction was detected between FLAG-PSI A and GST-PSI A (Figure 1D(a), green oval), suggesting homodimerization of PSI A at this pH. Western blot analysis further confirmed the presence of FLAG-PSI A in the pull-down sample at pH 7.4, reinforcing the observation of PSI A homodimerization (Figure 1D(b)). Notably, PSI B homodimerization was not detected in the pull-down assays (Figure 1C) using any of the employed techniques, including silver staining and Western blot analysis.

### 2.2. PSI Homo- and Heterodimerization Confirmed In Vivo by mbSUS

**Figure 2:**
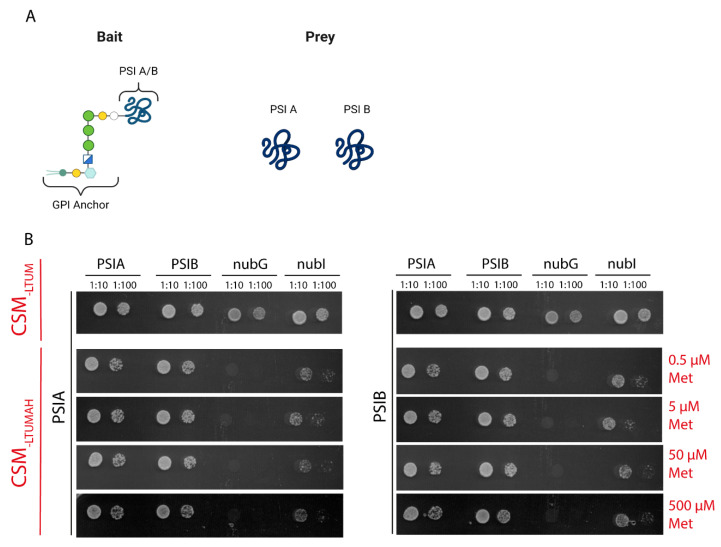
Split ubiquitin yeast two-hybrid assay. (**A**) Illustrations represent the bait and prey structures. The bait protein is fused to a typical GPI anchor [22]. (**B**) Yeast mating-based split-ubiquitin assay for interaction, including negative control (NubG) and positive control (NubI). Yeast diploids dropped at 1:10 and 1:100 dilutions spotted (**left** to **right**) on complete synthetic medium without Trp, Leu, Ura, and Met (CSM-LTUM) to verify mating; on CSM without Trp, Leu, Ura, Ade, His, and Met (CSM-LTUMAH) to verify adenine- and histidine-independent growth; and with Met additions to suppress bait expression.

To confirm the interactions between PSI A and PSI B in vivo, we employed the mating-based split-ubiquitin system (mbSUS), a highly sensitive and efficient alternative to the classical yeast two-hybrid system [21]. This assay was chosen to validate PSI–PSI interactions within a heterologous system, providing robust in vivo evidence. PSIs were fused to a glycosylphosphatidylinositol (GPI) anchor (Figure 2A) to facilitate detection, as no growth was observed in any of the media without this modification.

Yeast growth was observed on selective media containing varying concentrations of methionine (0, 5, 50, and 500 µM), which inhibits bait expression. PSI A–B NubG negative control mated yeast showed growth only at 0 µM methionine, as expected, with proliferation halting at higher methionine concentrations. In contrast, PSI A–B positive control NubI mated yeast proliferated across all methionine concentrations tested, indicating a strong and stable interaction. Furthermore, PSI–PSI mated yeast consistently proliferated even in the presence of 500 µM methionine, confirming that both homo- and heterodimerization of PSI A and PSI B occur robustly in yeast and are resistant to varying methionine concentrations.

These results provide strong evidence that PSI A and PSI B can form both homo- and heterodimers in a heterologous system, and that their interactions are stable and specific, supporting the hypothesis that PSI dimerization plays a key role in cellular function.

### 2.3. PSI–PSI Interactions Confirmed in Planta Using FRET-FLIM

To further validate the PSI–PSI interactions observed in earlier experiments, we employed FRET-FLIM in planta. This technique is highly reliable for detecting protein–protein interactions because the fluorescence lifetime is independent of fluorophore concentration, making it ideal for detecting close-range interactions even in varying concentration environments.

**Figure 3:**
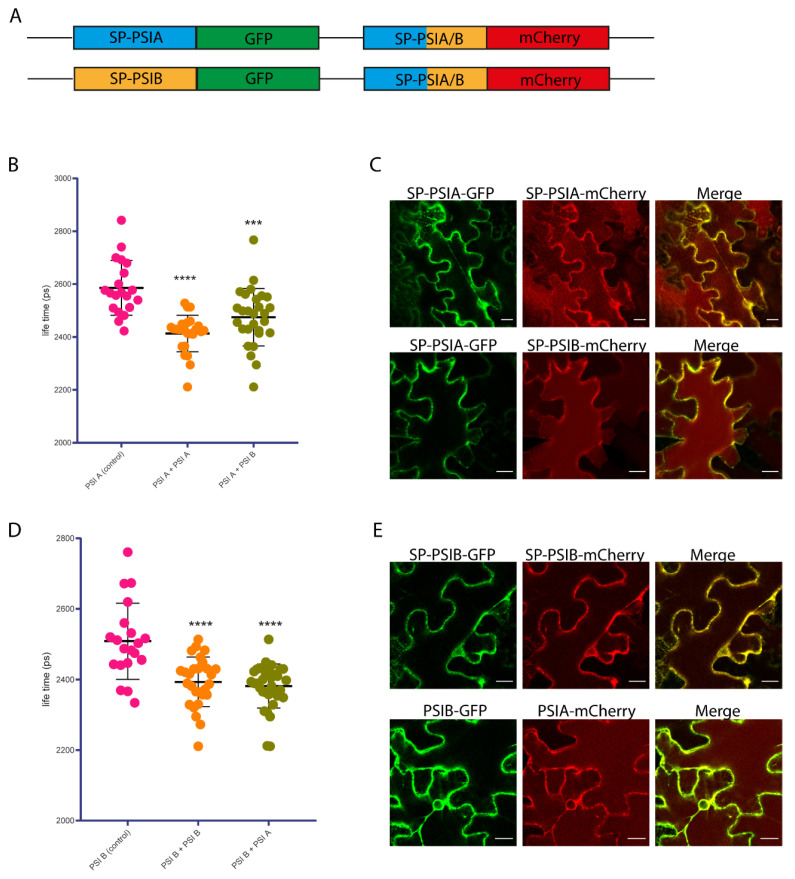
FRET-FLIM interaction assay. (**A**) Schematic representation of the fluorescent protein fusions performed in this study. Blue, yellow, green, and red rectangles represent SP-PSIA, SP-PSIB, green fluorescent protein (GFP), and red fluorescent protein (mCherry), respectively. (**B**) Mean lifetime graphic representation of PSI A—PSIA/B fluorescent protein pairs. Asterisks represent statistically significant differences in mean fluorescence lifetime with an α threshold of 0.05 and a 95% confidence interval (***, *p* < 0.0002; ****, *p* < 0.0001). (**C**) Subcellular localization of PSI A—PSIA/B fluorescent protein pairs. (**D**) Mean lifetime graphic representation of PSI B—PSI A/B fluorescent protein pairs. Asterisks represent statistically significant differences in mean fluorescence lifetime with an α threshold of 0.05 and a 95% confidence interval (****, *p* < 0.0001). (**E**) Subcellular localization of PSI B—PSIA/B fluorescent protein pairs. SP—signal peptide.

Using the pFRETgc-2in1-CC vector, PSI A and PSI B were cloned into the same vector backbone and fused to donor (eGFP) and receptor (mCherry) fluorophores, respectively (Figure 3A). The results showed a significant (*p* < 0.005) reduction in mean fluorescence lifetime compared to the control (PSI interaction with a gentamicin resistance gene), indicating that both PSI A and PSI B were close enough to allow for energy transfer between the fluorophores.

For the PSI A–PSI A interaction, a notable decrease in GFP fluorescence lifetime was detected, confirming homodimerization of PSI A (Figure 3B). Similarly, a significant reduction in lifetime was observed for the PSI A–PSI B interaction, supporting the formation of heterodimers between these two proteins. Imaging results further demonstrated that both PSIs co-localize across the cell (Figure 3C). In the vacuole, only PSI–mCherry fusions were detected due to the degradation of GFP in the acidic environment of the vacuole.

In the case of PSI B–PSI B interactions, there was again a statistically significant decrease in fluorescence lifetime (*p* < 0.005), suggesting homodimerization of PSI B (Figure 3D). As with PSI A, co-localization was observed across the cell (Figure 3E).

### 2.4. In Silico Analysis Revealed an Internal Loop in PSI with Potential for Membrane Binding

**Figure 4:**
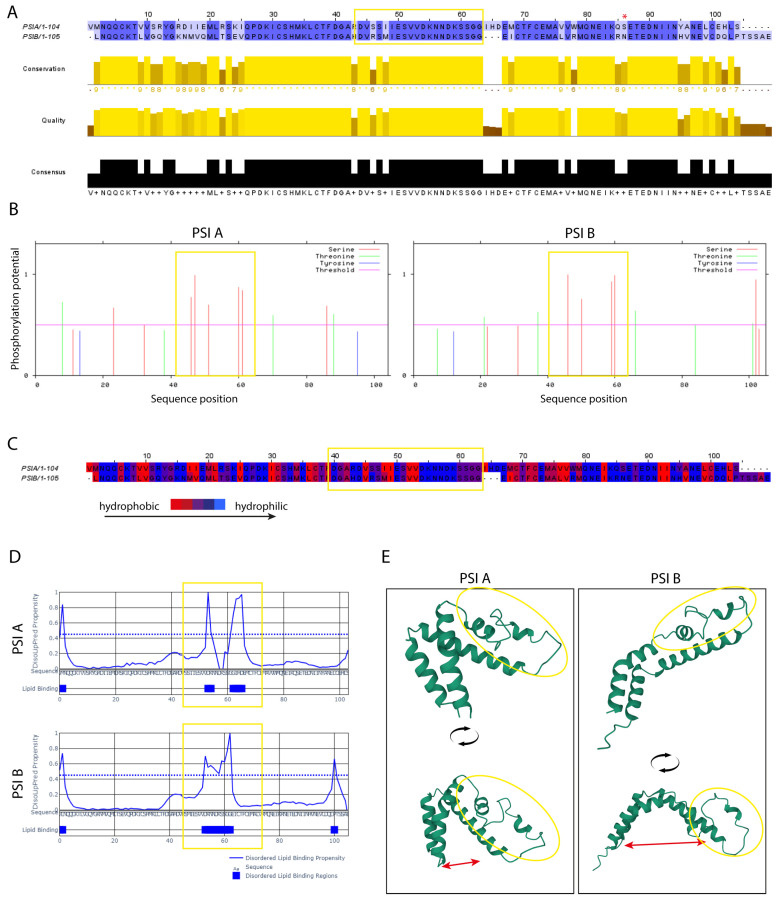
Bioinformatic analysis of the cardoon PSI A and PSI B amino acidic sequence. (**A**) Identification of conserved regions between PSI A and PSI B. Analysis performed with NetPhos 3.1 webtool (https://services.healthtech.dtu.dk/services/NetPhos-3.1/, accessed on 21 January 2024). Yellow zones represent conserved regions between PSI A and B while the red asterisk (*) represents a glycosylation site in PSI B. (**B**) Prediction of phosphorylation sites in both PSIs. Produced with Jalview webtool (https://www.jalview.org, accessed on 21 January 2024). (**C**) Analysis of the hydrophilic potential of PSI A and PSI B amino acid sequence. Produced with Jalview webtool (https://www.jalview.org/, accessed on 21 January 2024) (**D**) Lipid binding potential prediction of amino acid regions in cardoon PSI A and PSI B. Analysis done with DisoLipPred webtool (http://biomine.cs.vcu.edu/servers/DisoLipPred/, accessed on 21 January 2024). (**E**) Tertiary structure prediction of PSI A and PSI B. Double-edged red arrow is used to show that PSI A possesses a clustered tertiary structure while PSI B is a bit wider. Produced with AlphaFold webtool (https://alphafold.ebi.ac.uk/, accessed on 21 January 2024). Dashed lines represent the threshold value for putative lipid interaction detected by the software. In all figures, the yellow square represents an uncharacterized but conserved loop region found in both PSIs that has potential for lipid interaction and therefore may interact with membranes.

To further investigate the dimerization ability observed in our experimental assays, we turned to an in silico analysis of the amino acid sequences of PSI A and PSI B. Figure 4 summarizes the key findings from this analysis.

A sequence alignment between PSI A and PSI B revealed a high degree of conservation, particularly in regions associated with potential interaction sites (Figure 4A). The dark blue regions indicate areas of high sequence identity, which may be important for the formation of both homo- and heterodimers. Using NetPhos 3.0, we identified several putative phosphorylation sites—nine in PSI A and eight in PSI B—with most sites involving serine residues (Figure 4B). Interestingly, while not all phosphorylation sites were shared between the two PSIs, a cluster of conserved phosphorylation sites was identified in the central region of both PSIs (Figure 4B, yellow square).

Given the established role of PSIs in membrane interactions, we also examined the hydrophilic potential of the sequences, as lipid-binding proteins often contain hydrophilic regions interspersed with hydrophobic domains. Both PSI A and PSI B exhibited hydrophilic and hydrophobic patches, with several domains predicted to interact with lipids using the DisoLipPred web tool (Figure 4D). PSI A contains three putative lipid-binding regions, while PSI B contains four, suggesting both PSIs could interact with cellular membranes in specific regions.

Using the published StAP PSI structure as a model, we obtained the predicted structures of PSI A and PSI B (Figure 4E). Both structures resemble the characteristic PSI bending seen in StAP, although the angle between the two halves differs between PSI A and PSI B (Figure 4E, red arrows). Interestingly, the regions identified as important for phosphorylation, hydrophilic potential, and lipid-binding ability correspond to the loop where the PSIs bend (Figure 4E, yellow circle), a feature that may influence their dimerization and facilitate membrane interaction.

Overall, these bioinformatic analyses provide additional evidence supporting the dimerization ability of PSI A and PSI B, particularly through conserved regions and structural features that could facilitate their interaction and function in membrane-associated processes.

## 3. Discussion

### 3.1. PSI Homo- and Heterodimerization May Relate to Their Role and Mode of Action

The functions of the plant-specific insert (PSI), both as an independent entity and as part of the proenzyme, remain largely elusive. Previous studies have suggested that PSIs may exert different roles when forming dimers compared to their isolated monomeric forms [5]. Furthermore, their activities appear to be influenced by their ability to interact with membranes, in a manner dependent on both lipid content and pH [7,15]. Our study explored these dynamics by investigating the dimerization potential of PSI A and PSI B, particularly focusing on their ability to form homo- and heterodimers using a combination of in vitro, in vivo, and in silico approaches.

As an initial exploratory approach, we conducted in vitro pull-down assays to investigate potential interactions between PSIs. These assays suggested that pH conditions influenced dimer formation, with both PSI A–A and PSI A–B dimers detected at pH 6.8, while only PSI A homodimers persisted at pH 7.4. No interaction was observed between PSI B molecules at either pH. However, while these results offered preliminary insights into the dimerization dynamics, they should be considered secondary to the in vivo evidence provided by subsequent assays.

One likely explanation for the limited interaction observed for PSI B in the in vitro assays is the absence of glycosylation. PSIs expressed in bacteria lack N-glycosylation, a post-translational modification that has been shown to be crucial for PSI B’s proper function and transport [14]. The lack of glycosylation could destabilize PSI B’s ability to form dimers in vitro or the protein domain itself. In contrast, in vivo systems provide the necessary cellular machinery for post-translational modifications such as N-glycosylation, which likely stabilizes PSI B interactions. Therefore, the in vitro data, while informative, do not fully reflect the physiological behavior of PSI B and should be interpreted as exploratory.

More conclusive in vivo data were obtained through the mating-based split-ubiquitin system (mbSUS), which demonstrated robust and specific homo- and heterodimerization of PSI A and PSI B. These findings strongly suggest that PSI dimerization is stable under physiological conditions and likely occurs regardless of external pH variations, supporting the hypothesis that PSI interactions are key to their cellular function.

The FRET-FLIM assays further corroborated these results by confirming the proximity and interaction of PSI A and PSI B in planta. Not only did this technique provide direct evidence of PSI dimerization, but it also revealed that PSI B—unlike in vitro results —can form both homodimers and heterodimers in vivo. This suggests that post-translational modifications such as glycosylation play a key role in stabilizing these interactions, particularly for PSI B.

The results align with prior findings, such as those observed in recombinant, non-glycosylated PSI from spear thistle (*Cirsium vulgare*), where PSI was shown to form dimers at pH 7.5 in nonreducing conditions [3,4]. Additionally, these findings correspond with studies on potato PSI, where deviations from the monomer–dimer equilibrium were observed at similar pH values, reinforcing the notion of context-dependent PSI dimerization [4]. In contrast to our findings with PSI A and B, previous research on potato StAP PSI, which lacks a putative glycosylation site, has predominantly focused on dimerization independent of such modifications. These studies have helped establish a foundation for understanding PSI dimerization but may not fully capture the complexity observed in glycosylated PSIs from other plant systems, such as cardosins [5].

### 3.2. The Loop Region of the PSI as a Putative Membrane Interaction Domain

To better understand the experimental results and elucidate the molecular mechanisms behind PSI–PSI interactions, we performed a systematic bioinformatic analysis of PSI A and PSI B. Focusing on potential membrane interaction sites and post-translational modifications, we modeled the structures of PSI A and PSI B based on the StAP PSI structure (UniProt-M1BU17) [7]. These models revealed that both PSIs have a helical configuration with a prominent unstructured loop region located in the middle of the protein.

Further sequence analysis showed that the loop region is enriched with putative phosphorylation sites, hydrophilic residues, and regions with a high likelihood for protein–lipid interactions. These structural characteristics suggest that the loop may play a critical role in PSI-mediated membrane interactions. This is consistent with previous studies that highlighted the involvement of PSIs in membrane-related activities and their role as antimicrobial agents [6,9]. Such membrane interactions could make PSIs important components of plant defense mechanisms, where their ability to target or disrupt membranes may be crucial.

In addition to potential roles in membrane interactions, the loop region’s structural features could also play a role in vacuolar trafficking mechanisms. The sequence variations between PSI A and PSI B, along with intracellular pH gradients, may influence the distinct pathways that these PSIs follow. For instance, PSI A mediates an unconventional trafficking route, bypassing the Golgi apparatus, while PSI B follows the conventional ER–Golgi pathway [14]. The loop region, with its membrane-interacting potential, might contribute to these differences in trafficking by enabling PSI A to interact with membranes and vesicles early in the endomembrane system.

Based on our in silico analysis, we propose a working model where the loop region facilitates PSI interaction with membranes, assisting in their sorting between cellular compartments. For example, PSI A may form dimers early in the endomembrane system, allowing it to promote membrane budding and interact with unknown vesicles, enabling it to bypass the Golgi through an unconventional route [23]. Conversely, PSI B, which follows the traditional ER–Golgi pathway, might not require such interactions under neutral pH conditions, given the gradual decrease in pH along the endomembrane system.

While we have not yet fully elucidated the influence of pH on PSI-mediated transport, we propose that pH sensitivity may vary between PSIs, with the PSI A–A dimer potentially being more stable under basic pH conditions. This hypothesis aligns with our observations of PSI A homodimerization at pH 7.4, suggesting that pH could play a role in regulating dimer stability and function.

Additionally, it is plausible that the distinct pathways followed by PSI A and PSI B are related to their ability to dimerize and interact with membranes in pH-specific environments. PSI A may require these interactions to adapt to fluctuating pH levels along the endomembrane system, particularly as it bypasses the Golgi through an unconventional pathway. In contrast, PSI B, following the more traditional ER–Golgi route, may not need to adapt to such pH fluctuations, maintaining a more stable, pH-independent pathway.

### 3.3. Biological Significance of PSI Interactions and Aspartic Proteinases in Plant Physiology

Aspartic proteinases (APs) containing plant-specific inserts (PSIs) play critical roles in plant physiology by facilitating vacuolar sorting, membrane trafficking, and the modulation of cellular environments, especially under stress conditions [1,5]. The dimerization of PSIs, as demonstrated in this study, is not merely a mechanistic process but a vital aspect of how APs function in broader biological contexts.

In normal physiological conditions, PSIs mediate the correct intracellular transport of APs to vacuoles, which are key organelles involved in cellular homeostasis, storage, and degradation of macromolecules [10,14]. This function is crucial in tissues with high metabolic activity, such as flowers and seeds, where protein turnover and storage are essential for growth and reproduction [18]. PSIs, through their ability to interact with membranes and dimerize, likely fine-tune these processes by ensuring efficient vacuolar targeting, preventing premature protein degradation, and maintaining cellular proteostasis [4,15].

Under stress conditions, such as drought, salinity, or pathogen attack, aspartic proteinases, including those with PSIs, have been shown to be involved in plant defense mechanisms [6]. For example, the saposin-like domain found in PSIs exhibits membrane-disrupting properties that may contribute to antimicrobial activities, aiding in the plant’s defense against pathogens [9]. Additionally, the regulated dimerization of PSIs in response to environmental pH changes or glycosylation states might help plants adapt their intracellular transport mechanisms to fluctuating stress conditions [5]. In this context, the ability of PSIs to form homo- and heterodimers could play a pivotal role in modulating protein trafficking pathways, ensuring that proteolytic enzymes are correctly targeted during stress responses.

Moreover, recent studies have highlighted that the dimerization and membrane-binding capabilities of PSIs might also contribute to the plant’s ability to manage oxidative stress [6]. The regulation of vacuolar protease activity through PSI interactions could be essential for mitigating the effects of reactive oxygen species (ROS), which accumulate during various stress responses [5,9]. This ability to respond to stressors through modulated dimerization emphasizes the biological importance of PSIs in both normal cellular function and in defense mechanisms [11].

Overall, the interactions between PSIs, particularly their dimerization, are not only essential for vacuolar targeting but also likely contribute to the plant’s adaptive responses to environmental stressors. These findings underscore the broader biological significance of PSI-containing APs in plant development, stress adaptation, and overall homeostasis.

## 4. Materials and Methods

### 4.1. Plant Material and Growth Conditions

In this study, *Nicotiana tabacum* was used as a heterologous system to express cardosins’ PSIs. Wild-type *Nicotiana tabacum* plants were sown directly on fertilized substrate (Universal substrate, SYRO PLANT). All plants were grown under long day conditions (16 h light) at 24 °C with 50–60% relative humidity and light intensity at 180 μmole m^−2^ s.

### 4.2. Constructs Generation

For pull-down assays, PSI A and PSI B sequences were amplified by PCR with specific primers introducing either a FLAG or a 6x His tag sequence at the N-terminal and C-terminal portions, respectively (Supplementary Table S1). FLAG-PSI and PSI-6xHis sequences were cloned into the pET-21a(+), containing a 6× His tag, and pGEX-6p-1, containing a glutathione S-transferase (GST) tag, respectively, through Gibson assembly [24]. The expression vectors were transformed into *Escherichia coli* BL21 (DE3).

For mating-based split-ubiquitin (mbSUS) assays, PSI A and PSI B sequences were amplified by PCR with specific primers with adaptors for Gateway cloning (Supplementary Table S2). Purified fragments were cloned into the pDONR201 entry vector via a BP recombination reaction, as described above. The entry vectors were transferred into pMetYOst-Dest, and pNX35-Dest destination vectors [21] through an LR recombination reaction.

For FRET-FLIM analysis, PSI A and PSI B sequences were amplified by PCR with specific primers with adaptors for Gateway cloning (Supplementary Table S3). Purified PCR products were cloned into pDONR221 P1–P4 and pDONR221P3-P2 entry vectors (Invitrogen, Waltham, MA, USA) through a BP recombination reaction, as described by Grefen and Blatt [25]. The entry vectors were transferred into the pFRETgc-2in1-CC destination vector [26] through a 2in1 LR recombination reaction according to Grefen and Blatt [25]. A pDONR221P3-P2 containing the gentamycin resistance gene used as a control.

All constructs were confirmed by DNA sequencing (Eurofins Genomics, Luxembourg City, Luxembourg). Expression vectors for plant transformation were delivered into *Agrobacterium tumefaciens* GV3101, as described previously [27].

### 4.3. PSI Expression and Purification

*Escherichia coli* BL21 (DE3) cells transformed with either pET-21a::FLAG-PSIA-6xHis, pET-21a::FLAG-PSIB-6xHis, pGEX-6p-1::GST-PSIA-6xHis, or pGEX-6p-1::GST-PSIB-6xHis were incubated in LB medium with 100 μg/mL ampicillin overnight at 37 °C with shaking (200 rpm) to obtain the starter cultures. Starter cultures were transferred into 2 L LB medium with 100 μg/mL ampicillin and incubated for 4 h at 30 °C with shaking. The temperature was then lowered to 23 °C for 1 h, and 125 µM IPTG was added to induce cell expression. After overnight growth at 23 °C with shaking, cells were harvested by centrifugation at 4000× *g* for 15 min at 4 °C. The pellet was resuspended in 10 mL of lysis buffer [50 mM Tris-HCl pH 8, 200 mM NaCl, 10 mM Imidazole, 1 mM DTT, and 0.1% (*v*/*v*) Tween-20], with 125 µL of cOmplete^TM^, Mini, EDTA-free Protease Inhibitor Cocktail (Roche^®^ Life Science Products, Basel, Switzerland) and 1 mg/mL of lysozyme (Roche^®^ Life Science Products, Basel, Switzerland). The lysate was incubated on ice for 15 min, and sonicated 2 times for 30 s, using three different outputs (10, 15, and 20) at 1 min intervals (XL-2000, QSONICA, Newtown, CT, USA). The lysate was centrifuged at 35000× *g* for 45 min at 4 °C. Cleared lysate was incubated with 750 µL of HisPur^TM^ Ni-NTA Superflow Agarose (Thermo Fisher Scientific, Waltham, MA, USA) for 1 h at 4 °C and centrifuged at 700× *g* for 3 min. The supernatant was removed, and the resin was washed for 5 min with 10 mL of ice-cold wash buffer [25 mM Tris-HCl, pH 8, 200 mM NaCl, and 25 mM imidazole]. The washing steps were then repeated 3 times. The proteins were batch-eluted with 1 mL of elution buffer [50 mM Tris-HCl pH 8, 150 mM NaCl, and 250 mM imidazole] for 5 min. Intermediated protein fractions were stored during the purification process for control purposes—non-induced, induced, lysate, soluble, flow through, bound to resin, washed resin, elution fractions, and resin after elution fractions.

### 4.4. In Vitro Pull-Down Assays

Purified GST-PSI A/B-6xHis (6 µg) was combined with FLAG-PSI A/B-6xHis (2 µg) in a final volume of 100 µL of pull-down buffer [50 mM Hepes, 50 mM NaCl, 0.1% Triton X-100, and 5 mM DTT] prepared at pH 6.8 and 7.4. GST-tagged proteins were pulled using 30 µL of Pierce^TM^ Glutathione Agarose beads (Thermo Fisher Scientific, Waltham, MA, USA), as described by the manufacturer. The reactions were incubated for 1 h at 4 °C on an overhead rotator. The mixture was centrifuged at 500× *g* for 15 s, the supernatant was removed, and the resin was washed for 3 min with 500 µL of pull-down buffer. This washing step was repeated 3 times. After the last wash, 20 µL of 1X SDS-PAGE sample loading buffer were added and the mixture was heated at 95 °C for 5 min. A positive control to assess the binding of the GST-PSI A/B-6xHis purified proteins to the beads and a negative control to ensure that the putative interactor did not bind to the beads were prepared at the same time.

### 4.5. SDS-PAGE and Western Blot Analysis

Denatured protein fractions were analyzed by SDS-PAGE using a 15% polyacrylamide gel. Protein samples were prepared by adding 1X SDS-PAGE sample loading buffer [0.125 M Tris-HCl pH 6.8, 15% (*v*/*v*) glycerol, 4% (*w*/*v*) SDS, 100 mM DTT, and 0.02% (*v*/*v*) bromophenol blue]. The samples were incubated at 95 °C for 5 min and spun for 2 min immediately before gel application. Five microliters of each protein fraction were loaded onto the gel, and PageRuler Prestained Protein Ladder (Thermo Fisher Scientific, Waltham, MA, USA) was used as a protein molecular weight marker. After electrophoresis, polyacrylamide gels were stained using either BioRad Silver Stain Kit (BioRad, Hercules, CA, USA) or BlueSafe Coomassie staining (NZYtech, Lisbon, Portugal) according to the manufacturer’s instructions.

For Western blot analysis, protein samples were transferred to a nitrocellulose membrane (Amersham Protran 0.2 µm NC, GE healthcare, Chicago, IL, USA) in transfer buffer [25 mM Tris, 192 mM glycine, and 20% (*v*/*v*) methanol], using the Trans-blot turbo system (BioRad, Hercules, CA, USA). Nonspecific binding was blocked with a blocking solution [Tris-buffered saline (TBS) (50 mM Tris and 200 mM NaCl) with 0.1% (*v*/*v*) Tween 20 (TBS-T) supplemented with 5% (*w*/*v*) skim milk] for 1 h at room temperature. The primary antibody (DYKDDDDK Tag Monoclonal Antibody, Thermo Fisher Scientific) was diluted 1:1000 in blocking solution and incubated with the membrane overnight at 4 °C with constant shaking. Afterwards, the membrane was washed with TBS-T for 10 min and then two times for 5 min. The membrane was incubated for 1 h at room temperature with the secondary antibody (HRP IgG, Sigma-Aldrich, St. Louis, MO, USA) diluted 1:5000 in blocking buffer. The membrane was washed as previously described, the bands were revealed using the Clarity Western ECL Substrate (Bio-Rad, Chigago, IL, USA) according to the manufacturer’s instructions, and the signal was detected by chemiluminescence using a ChemiDoc XRS+ System (Bio-Rad) running Image Lab (Bio-Rad).

### 4.6. Mating-Based Split-Ubiquitin (mbSUS) Assays

Haploid yeast (*Saccharomyces cerevisiae*) strains, THY.AP4 [MATα ura3 leu2 lexA::lacZ::trp1 lexA::HIS3 lexA::ADE2] for bait and THY.AP5 [MATα URA3 leu2 trp1 his3 loxP::ade2] for prey [21], respectively, were transformed with pMetYOst-Dest or pNX35-Dest constructs carrying PSI A or PSI B. After transformation, THY.AP4 contained the fusion protein with C-terminal Y-CubPLV (bait), whereas THY.AP5 contained the fusion protein with N-terminal NubG-X (prey). A mating-based screening assay was performed to assess the interactions between Y-CubPLV and NubG-X fusion proteins. Ten to fifteen yeast colonies of transformed THY.AP4 and THY.AP5 were inoculated into selective Complete Synthetic Media (CSM) [21] lacking leucine (Leu), and methionine (Met) (CSM-LM) and CSM lacking tryptophan (Trp), uracil (Ura), and Met (CSM-TUM), respectively, and incubated at 28 °C overnight with shaking (180 rpm). Yeast cells were harvested by centrifugation at 2000× *g* for 1 min at room temperature and resuspended in yeast extract peptone dextrose (YPD) medium. Yeast mating was performed in sterile PCR tubes by mixing equal aliquots of yeast carrying the bait and prey constructs. Aliquots (5 μL) were dropped onto YPD plates and incubated at 28 °C overnight. Mated yeasts were transferred from YPD onto CSM lacking Leu, Trp, Ura, and Met (CSM-LTUM) plates and incubated at 28 °C for 2–3 days. Diploid colonies were inoculated in liquid CSM-LTUM medium and grown at 28 °C overnight with shaking. The remaining mated cells were harvested by centrifugation at 2000× *g* for 1 min at room temperature and resuspended in sterile water to an OD600 of 1.0 and 0.1. Each dilution (5 μL) was dropped on CSM lacking Leu, Trp, Ura, Met, adenine (Ade), and His (CSM-LTUMAH) plates with different concentrations of Met (0.5, 5, 50, and 500 µM) to increase the signal-to-noise ratio. Plates were incubated at 28 °C, and images were taken after 3 days. Yeast cells were also dropped onto CSM-LTUM control plates to confirm mating efficiency and cell density, and growth was imaged after 24 h at 28 °C. Two controls were performed, a positive control with diploid yeast containing NubI (wild-type) as prey, and a negative control with diploid yeast containing NubG as prey. Images of the interaction plates were obtained using a flatbed scanner. Each interaction pair was tested three times at each Met concentration [21].

### 4.7. Plant Transformation

*Agrobacterium tumefaciens* GV3101 was used to transiently transform N. tabacum with 2in1 FRET-FLIM constructs through syringe-mediated infiltration as described by Sparkes et al. [27]. Bacterial cells were harvested, washed in sterile water, and resuspended in ice-cold infiltration medium (10 mM MgCl_2_, 10 mM MES-KOH pH 5.6, and 150 μM acetosyringone) to an OD600 of 0.3. After an incubation time of 3 h on ice, leaves of 3- to 4-week-old N. tabacum plants were infiltrated. Lower epidermis of infiltrated leaves was analyzed through confocal laser scanning microscopy (CLSM) analysis 48 h post-infiltration.

### 4.8. FRET-FLIM Analysis

FRET-FLIM experiments were performed using a Leica TCS SP8 confocal microscope (Leica Microsystems, Wetzlar, Germany) equipped with a FLIM unit (PicoQuant, Berlin, Germany). Images were acquired using a 20× water immersion objective (Leica Microsystems, Wetzlar, Germany) using LAS-X version 3.5 software (Leica Microsystems, Wetzlar, Germany). The 488 nm laser line was used for excitation of mEGFP, whereas the 561 nm line was used to excite mCherry. Emission data were collected at 495–530 nm for mEGFP and 580–630 nm for mCherry. For FLIM measurements, N. tabacum cells were excited with a 470 nm pulsed laser (LDH-P-C-470), and emission was detected with a FLIM-compatible photomultiplier tube (PMT) from 495 to 530 nm by time-correlated single-photon counting using a Picoharp 300 module (PicoQuant, Berlin, Germany). FLIM measurements were performed on 20–30 cells derived from three independent infiltrations. Each time-correlated single-photon counting histogram was re-convoluted with a corresponding instrument response function and fitted against a monoexponential decay function to determine the mean GFP fluorescence lifetime in each assay. The average GFP fluorescence lifetimes were calculated using FLIMfit software version 5.1.1., developed at Imperial College London.

### 4.9. Bioinformatics

To identify conserved regions between PSI A and PSI B, NetPhos 3.1 webtool (https://services.healthtech.dtu.dk/services/NetPhos-3.1/, accessed on 21 January 2024) was used. Then, Jalview was employed (https://www.jalview.org, accessed on 21 January 2024) to assess putative phosphorylation sites present in the amino acid sequence of both PSIs, emphasizing three relevant phosphorylation-related amino acids—serine, threonine, and tyrosine. Jalview was also used to analyze the hydrophilic potential of PSI A and PSI B amino acid sequence. The tertiary structure prediction of PSI A and PSI B was performed with AlphaFold (https://alphafold.ebi.ac.uk/, accessed on 21 January 2024). Finally, DisoLipPred webtool (http://biomine.cs.vcu.edu/servers/DisoLipPred/, accessed on 21 January 2024) was used to study the lipid binding potential of amino acid regions in PSI A and PSI B.

## 5. Conclusions

This study has provided novel insights into the dimerization dynamics of plant-specific inserts (PSIs) in Cynara cardunculus, revealing complex molecular interactions with significant functional implications for plant biology. Through a combination of biochemical and bioinformatic analyses, we have demonstrated that PSI A and PSI B form stable homo- and heterodimers. In vitro assays suggested pH-dependent dimerization while robust in vivo interactions were confirmed using mating-based split-ubiquitin (mbSUS) and FRET-FLIM techniques, emphasizing the physiological relevance of PSI dimerization within cellular environments. The identification of key phosphorylation sites and lipid-binding regions supports the hypothesis that PSIs interact with membranes in a dimerization-dependent manner. Phosphorylation could regulate PSI dimerization, influencing their ability to bind membranes, while the presence of lipid-binding regions suggests a direct role in membrane interactions. Together, these factors likely influence PSI function, particularly in processes like protein trafficking and intracellular transport. Additionally, the methodological advancements employed in this study, particularly the mbSUS and FRET-FLIM assays, have allowed for us to explore PSI interactions with high specificity, offering valuable tools for studying protein dynamics in plant systems. This work provides a strong foundation for future research into PSI interactions with membranes, lipids, and other cellular components. It underscores the importance of PSIs in maintaining cellular balance, particularly through protein sorting and membrane trafficking. Future studies should focus on the broader implications of PSI dimerization for plant homeostasis, investigating how these interactions contribute to intracellular transport and react to environmental changes. In particular, the loop region of PSIs warrants further investigation, as its role in membrane interactions and dimer formation could be key to understanding PSI function. Continued research will be essential to fully understand the role of PSIs in plant cellular processes and their broader significance

## Data Availability

The original contributions presented in the study are included in the article/Supplementary Material. Further inquiries can be directed to the corresponding author.

## Supplementary Materials

The following supporting information can be downloaded at: https://www.mdpi.com/article/10.3390/molecules29215139/s1, Figure S1: SDS-PAGE analysis of PSI A and B purifications. Coomassie blue-stained 15% SDS-PAGE gel of purified fractions obtained during His-tagged GST-PSI A (**A**), GST-PSI B (**B**), FLAG-PSI A (**C**), and FLAG-PSI B (**D**) purification. Red arrows indicate the bands of the expected size. MW: Molecular Weight (PageRuler^TM^ Prestained Protein Ladder, Thermo Scientific); NI: non-induced fraction; I: induced fraction; L: lysate fraction; SF: soluble fraction; FT: flow-through fraction; BR: bound to resin fraction; WR: washed resin fraction; E1–E5: elution fractions; RE: resin after elution fraction. kDa: kilodaltons; Table S1: List of primers used in Gibson assembly for pull-down assays; Table S2: List of primers used in Gateway cloning for FRET-FLIM analysis; Table S3: List of primers used in Gateway cloning for mating-based split-ubiquitin (mbSUS) assays.
