# Supplementary material for: Interaction Dynamics of Plant-Specific Insert Domains from Cynara cardunculus: A Study of Homo- and Heterodimer Formation
Source: Molecules. 2024 Oct 30;29(21):5139. doi: 10.3390/molecules29215139 (PMC11547502; doi:10.3390/molecules29215139)
Supplement: Supplementary file 1 [file molecules-29-05139-s001.zip › supplementary material.pdf]

**Table S1**. **List of** primers used in Gibson assembly for pull-down assays.

| Fragment | Primer | SEQUENCE (5'-3') |
|---|---|---|
| **FLAG-PSI A** | Fw | AACACTTATCCTGTGTCGACAAGCTTGCGG |
| | Rev | CCGCAAGCTTGTCGACACAGGATAAGTGTT |
| FLAG-PSI B | Fw | CTTCAAGTGCAGAATGTGTCGACAAGCTTG |
| | Rev | CAAGCTTGTCGACACATTCTGCACTTGAAG |
| GST-PSI A | Fw | AAAAAAGGATCCGTCATGAACCAGCAATGCAA |
| | Rev | AAAAAAGAATTCGCTTTGTTAGCAGCCGGATC |
| GST-PSI B | Fw | AAAAAAGGATCCTTAAACCAACAATGCAAAAC |
| | Rev | AAAAAAGAATTCGCTTTGTTAGCAGCCGGATC |

**Table S2.** List of primers used in Gateway cloning for **mating based split-ubiquitin (mbSUS)** assays.

| Fragment | Primer | Sequence (5'-3') |
|---|---|---|
| PSI A | Fw | GGGGACAAGTTTGTACAAAAAAGCAGGCTTAATGAAG ACTAATCTTTTTCTCTTTCTCAT |
| | Rev | GGGGACCACTTTGTACAAGAAAGCTGGGTTGGATAA GTGTTCACACAACTCGTTGG |
| PSI B | Fw | GGGGACAAGTTTGTACAAAAAAGCAGGCTTAATGAAG ACTAATCTTTTTCTCTTTCTCAT |
| | Rev | GGGGACCACTTTGTACAAGAAAGCTGGGTTTTCTGCA CTTGAAGTGGGTAACTGAT |

**Table S3.** List of primers used in Gateway cloning for FRET-FLIM analysis.

| Vector | Fragment | Primer | Sequence (5'-3') |
|---|---|---|---|
| **pDONR 221 P1-P4** | PSI A | Fw | GGGGACAAGTTTGTACAAAAAAGCAGGCTATGAAGACTAATCTTTTTCTCT |
| **pDONR 221 P1-P4** | PSI A | Rev | GGGGACAACTTTGTATAGAAAAGTTGGGTGGGATAAGTGTTCACACAACTCGT |
| **pDONR 221 P1-P4** | PSI B | Fw | GGGGACAAGTTTGTACAAAAAAGCAGGCTATGAAGACTAATCTTTTTCTCT |
| **pDONR 221 P1-P4** | PSI B | Rev | GGGGACAACTTTGTATAGAAAAGTTGGGTGTTCTGCACTTGAAGTGGGTA |
| pDONR 221 P2-P3 | PSI A | Fw | GGGGACAACTTTGTATAATAAAGTTGCCATGAAGACTAATCTTTTTCTCT |
| pDONR 221 P2-P3 | PSI A | Rev | GGGGACCACTTTGTACAAGAAAGCTGGGTAGGATAAGTGTTCACACAACTCGT |
| pDONR 221 P2-P3 | PSI B | Fw | GGGGACAACTTTGTATAATAAAGTTGCCATGAAGACTAATCTTTTTCTCT |
| pDONR 221 P2-P3 | PSI B | Rev | GGGGACCACTTTGTACAAGAAAGCTGGGTATTCTGCACTTGAAGTGGGTA |
