# Supplementary figures and images for: Interaction Dynamics of Plant-Specific Insert Domains from Cynara cardunculus: A Study of Homo- and Heterodimer Formation
Source: Molecules. 2024 Oct 30;29(21):5139. doi: 10.3390/molecules29215139 (PMC11547502; doi:10.3390/molecules29215139)

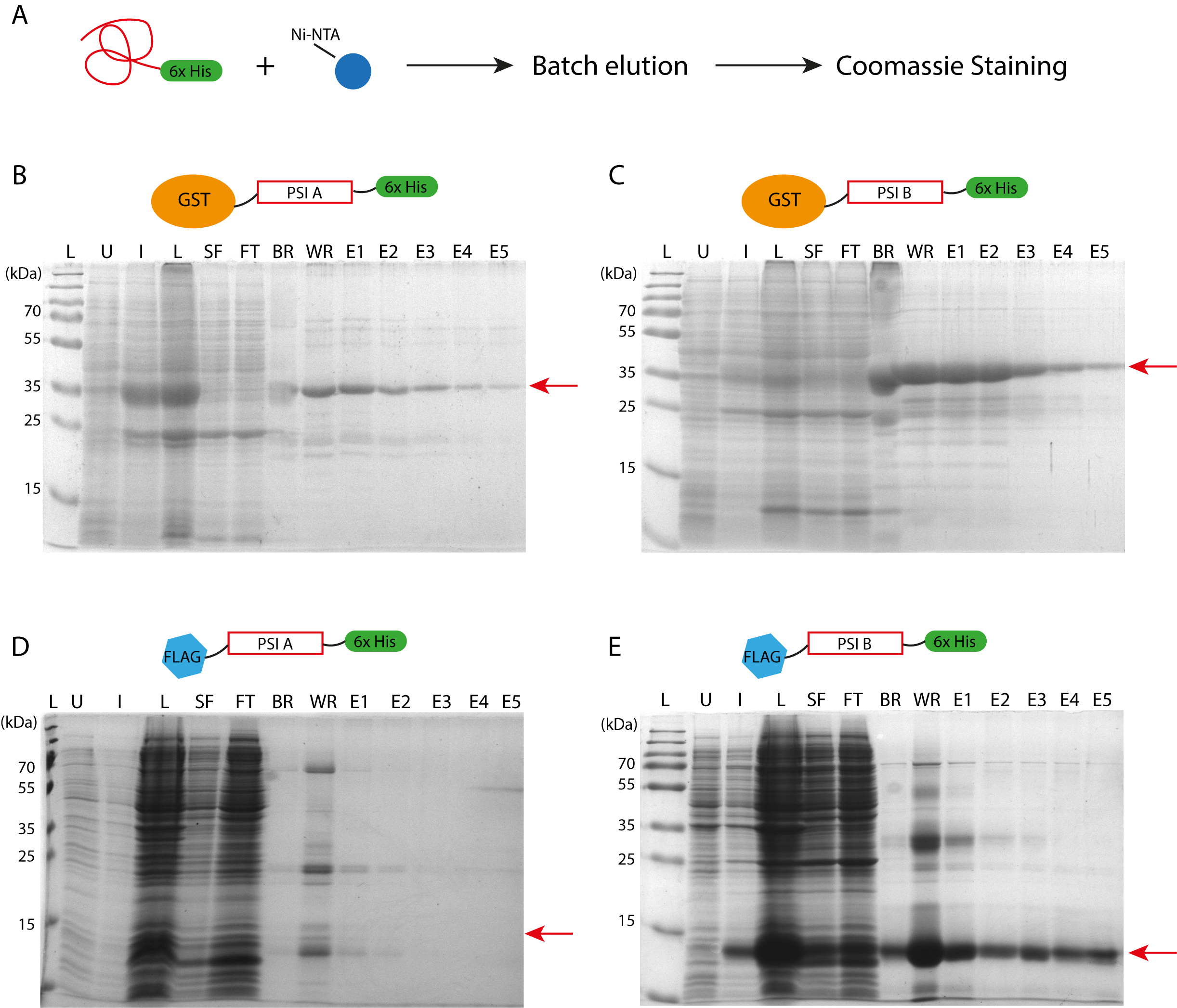

Supplement: Supplementary file 1 [file molecules-29-05139-s001.zip › Supplementary Figure S1.tif]
